# One-step selective hydroxylation of benzene to phenol with hydrogen peroxide catalysed by copper complexes incorporated into mesoporous silica–alumina†
†Electronic supplementary information (ESI) available: Cyclic voltammogram (Fig. S1), time profiles of phenol, *p*-benzoquinone, H_2_O_2_, spin or benzene (Fig. S2–S5 and S9 and S10), DFT results (Fig. S6 and Tables S1 and S2), UV-Vis absorption spectra (Fig. S7) and UV-Vis DRS (Fig. S8). See DOI: 10.1039/c5sc04312c


**DOI:** 10.1039/c5sc04312c

**Published:** 2016-01-05

**Authors:** Mihoko Yamada, Kenneth D. Karlin, Shunichi Fukuzumi

**Affiliations:** a Department of Material and Life Science , Graduate School of Engineering , Osaka University , ALCA and SENTAN , Japan Science and Technology (JST) , Suita , Osaka 565-0871 , Japan . Email: fukuzumi@chem.eng.osaka-u.ac.jp; b Department of Chemistry , The Johns Hopkins University , Baltimore , Maryland 21218 , USA . Email: karlin@jhu.edu; c Department of Chemistry and Nano Science , Ewha Womans University , Seoul 120-750 , Korea; d Faculty of Science and Engineering , Meijo University , ALCA and SENTAN , Japan Science and Technology Agency (JST) , Nagoya , Aichi 468-0073 , Japan

## Abstract

Benzene was hydroxylated with hydrogen peroxide (H_2_O_2_) in the presence of catalytic amounts of copper complexes in acetone to yield phenol at 298 K. At higher temperatures, phenol was further hydroxylated with H_2_O_2_ by catalysis of copper complexes to yield *p*-benzoquinone. The kinetic study revealed that the rate was proportional to concentrations of benzene and H_2_O_2_, but to the square root of the concentration of a copper(ii) complex ([Cu(tmpa)]^2+^: tmpa = tris(2-pyridylmethyl)amine). The addition of a spin trapping reagent resulted in formation of a spin adduct of hydroperoxyl radical (HO_2_˙), as observed by EPR spectroscopy, inhibiting phenol formation. HO_2_˙ produced by the reaction of [Cu(tmpa)]^2+^ with H_2_O_2_ acts as a chain carrier for the radical chain reactions for formation of phenol. When [Cu(tmpa)]^2+^ was incorporated into mesoporous silica–alumina (Al-MCM-41) by a cation exchange reaction, the selectivity for production of phenol was much enhanced by prevention of hydroxylation of phenol, which was not adsorbed to Al-MCM-41. The high durability with a turnover number of 4320 for the hydroxylation of benzene to phenol with H_2_O_2_ was achieved using [Cu(tmpa)]^2+^ incorporated into Al-MCM-41 as an efficient and selective catalyst.

## Abstract 2

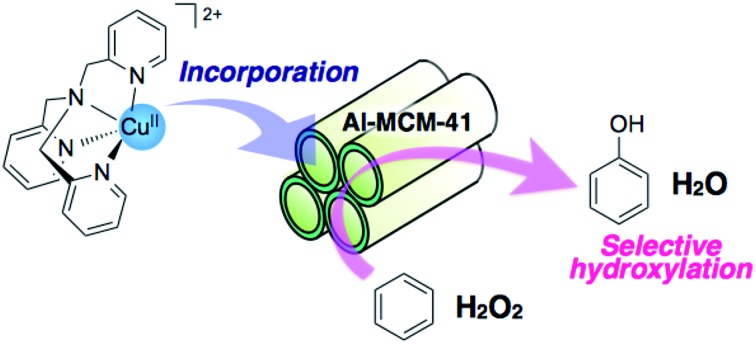
One-step hydroxylation of benzene with hydrogen peroxide to produce phenol catalyzed by a copper(ii) complex.

## Introduction

Phenol is one of the most important basic chemicals, widely used as a source for resins, fibres, and other various organic materials, being produced in industry by the three-step cumene process, which requires high energy consumption due to high-pressure conditions and produces acetone as a coproduct *via* cumene hydroperoxide.^1^,^2^ Thus, much effort has been devoted to develop a one-step hydroxylation of benzene to phenol using different oxidants such as N_2_O,^3^ O_2_ with reductants^4^–^9^ and H_2_O_2_.^10^–^23^ Among these oxidants, H_2_O_2_ has a clear advantage from the viewpoint of an environmentally benign green process and economical efficiency because of relatively mild reaction conditions for catalytic hydroxylation of benzene.^10^–^23^ However, selective hydroxylation of benzene to phenol with H_2_O_2_ has been difficult because phenol is normally easily over-oxidised to yield products such as *p*-benzoquinone.^10^–^22^ Although selective hydroxylation of benzene to phenol without over-oxidation has been achieved in some cases, the catalytic mechanism of hydroxylation of benzene to phenol has yet to be clarified,^24^–^26^ except for some cases of photocatalytic hydroxylation of benzene to phenol.^23^ Extensive efforts have been devoted to enhance the product selectivity and catalytic activity with heterogeneous catalysts, including metal complex catalysts incorporated into a mesoporous material. For instance, it has been reported that a manganese complex incorporated into a mesoporous material Al-MCM-41 exhibits a higher performance compared to that of the complex in a homogeneous solution because of the stabilisation of catalytically active species and prevention of phenol over-oxidation due to the highly acidic nature in Al-MCM-41.^26^

We report herein the one-step selective hydroxylation of benzene to phenol with H_2_O_2_ catalysed by a copper(ii) complex ([Cu(tmpa)]^2+^: tmpa = tris(2-pyridylmethyl)amine) incorporated into mesoporous silica–alumina in acetone at 298 K. The mechanism is clarified by a kinetic study and by detection of a reactive radical intermediate using a spin trap. The incorporation of the [Cu(tmpa)]^2+^ into mesoporous silica–alumina resulted in significant improvement in the selectivity and the durability of the catalyst.

## Results and discussion

### Hydroxylation of benzene to phenol with H_2_O_2_ catalysed by Cu(ii) complexes

**Fig. 1:**
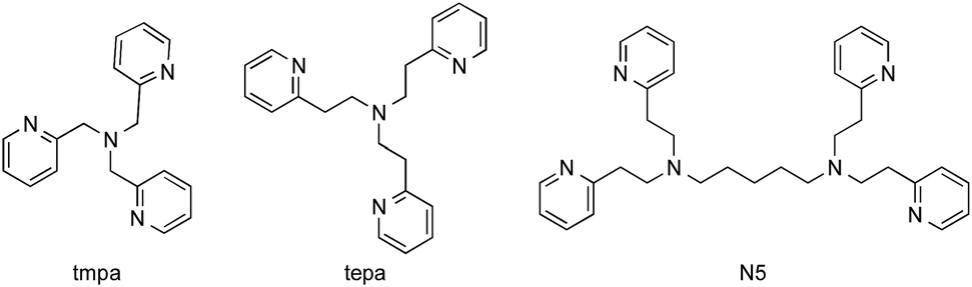
Chemical structures of ligands tmpa, tepa and N5.

**Fig. 2:**
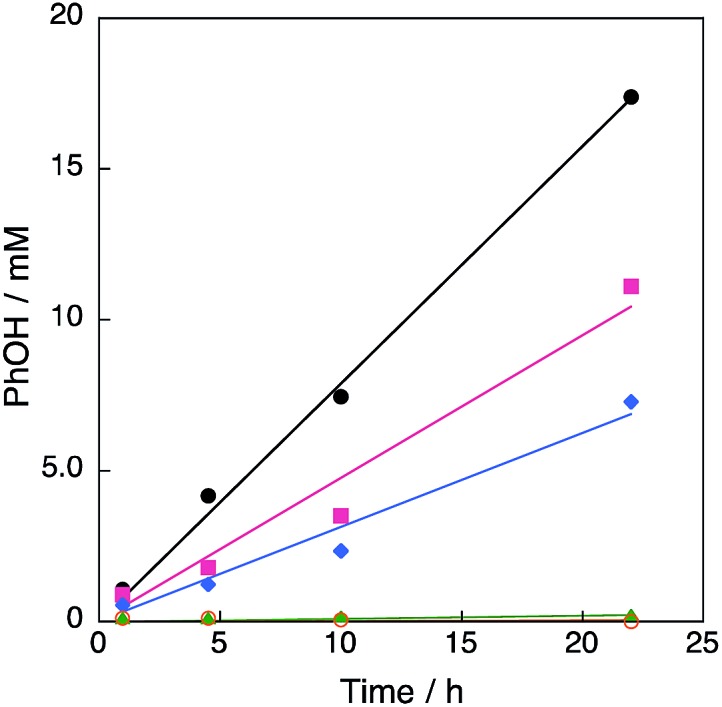
Time profiles of formation of phenol in the hydroxylation of benzene with 30 wt% aqueous H_2_O_2_ and a catalytic amount of **1** ([[Cu^II^(tmpa)]^2+^] = 67 μM, black circle), [Cu^II^(tepa)(ClO_4_)]ClO_4_ ([[Cu^II^(tepa)]^2+^] = 74 μM, pink square), [Cu^II^_2_(N5)(H_2_O)_2_](NO_3_)_4_ ([[Cu^II^_2_(N5)]^4+^] = 65 μM, blue diamond), Cu(ClO_4_)·6H_2_O (Cu^2+^ = 57 μM, green triangle) and no catalyst (orange open circle) in acetone at 303 K; [C_6_H_6_] = 2.1 M, [H_2_O_2_] = 2.1 M (4.75 mL).

A series of Cu(ii) complexes, [Cu^II^(tmpa)(CH_3_CN)](ClO_4_)_2_, [Cu^II^_2_(N5)(H_2_O)_2_](NO_3_)_4_ (N5 = –(CH_2_)_5_– linked bis[2-(2-pyridyl)ethyl]amine) and [Cu^II^(tepa)(ClO_4_)]ClO_4_ (tepa = tris(2-pyridylethyl)amine) were all found to catalyse hydroxylation of benzene with H_2_O_2_ to produce phenol (Fig. 1).^27^ The time profiles for these reactions using a catalytic amount of Cu(ii) complexes in acetone at 298 K are shown in Fig. 2. The most reactive Cu(ii) complex was [Cu^II^(tmpa)(CH_3_CN)](ClO_4_)_2_, which has the most negative one-electron reduction potential (*E*_red_ = –0.01 V *vs.* SCE)^28^ compared with those of [Cu^II^_2_(N5)(H_2_O)_2_](NO_3_)_4_ (*E*_red_*vs.* SCE = 0.27 V) and [Cu^II^(tepa)(ClO_4_)]ClO_4_ (*E*_red_*vs.* SCE = 0.44 V),^29^,^30^ as shown in Fig. S1 in ESI.† It should be noted that Cu^II^(ClO_4_)_2_ with no ligand exhibited virtually no catalytic activity. Thus, the tmpa ligand plays an essential role in the catalytic activity.

When acetone was replaced by strongly coordinating solvents, such as DMF and DMSO, no catalytic hydroxylation of benzene with H_2_O_2_ occurred using [Cu^II^(tmpa)(CH_3_CN)](ClO_4_)_2_ (**1**) as the catalyst (see Fig. S2 in ESI†). Thus, a coordination site is required to exhibit the catalytic activity. In acetonitrile, **1**-catalysed hydroxylation of benzene with H_2_O_2_ occurred to produce phenol. However, *p*-benzoquinone was also formed as a byproduct, which may be produced *via* oxidation of hydroquinone. In MeOH, the catalytic rate of hydroxylation of benzene with H_2_O_2_ was slower than those in acetone and acetonitrile. Thus, we have chosen acetone as a suitable solvent for mechanistic studies.

**Fig. 3:**
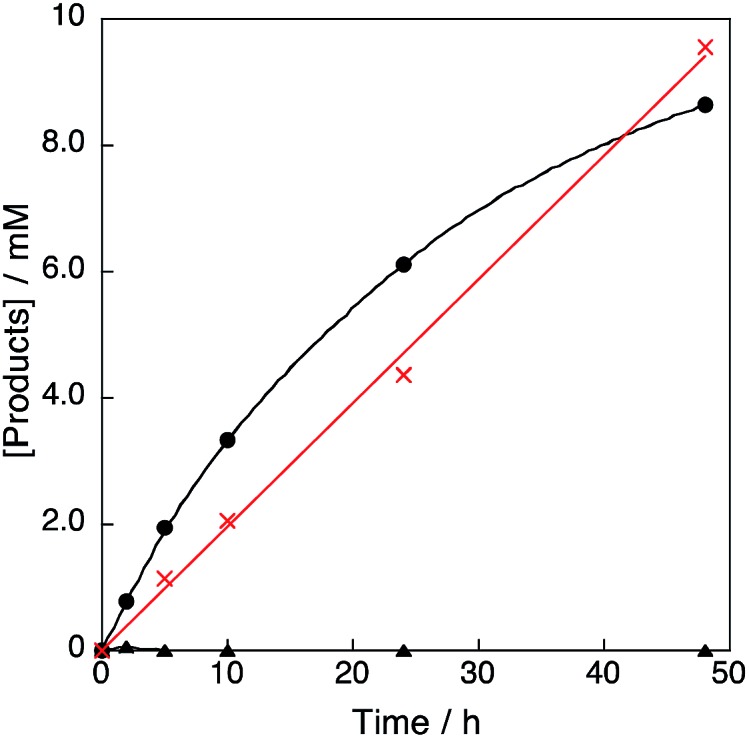
Time profiles of formation of phenol (black circle) and *p*-benzoquinone (black triangle) in the hydroxylation of benzene, and formation of *p*-benzoquinone (red cross) in the oxidation of phenol with 30 wt% aqueous H_2_O_2_ catalysed by **1** in acetone at 298 K; [C_6_H_6_] or [PhOH] = 2.1 M, [H_2_O_2_] = 2.1 M, [[Cu^II^(tmpa)]^2+^] = 67 μM in acetone (4.75 mL).

The products in the reaction of benzene with H_2_O_2_ and the Cu(ii) complex catalyst **1** were examined by GC-MS measurements. When a large concentration of benzene (2.1 M) was used for the **1**-catalysed reaction of benzene with H_2_O_2_, phenol was selectively produced without formation of *p*-benzoquinone at the initial stage of the reaction (black circle in Fig. 3). When benzene was replaced by phenol, however, *p*-benzoquinone was produced with a comparable rate as that for phenol production from benzene (red cross in Fig. 3).

The stoichiometry of the catalytic hydroxylation of benzene with H_2_O_2_ is given by eqn (1). The amount of reacted H_2_O_2_ was determined by the titration with Ti–TPyP (oxo[5,10,15,20-tetra(4-pyridyl)porphyrinato]titanium(iv);^31^ see Experimental section and Fig. S3 in ESI†). The ratio of the reacted H_2_O_2_ to phenol was determined to be nearly 3 : 1, indicating that H_2_O_2_ decomposed in competition with the catalytic hydroxylation of benzene to phenol.

1 C_6_H_6_ + H_2_O_2_ → C_6_H_5_OH + H_2_O

### Kinetics of catalytic hydroxylation of benzene to phenol with H_2_O_2_ and **1**

**Fig. 4:**
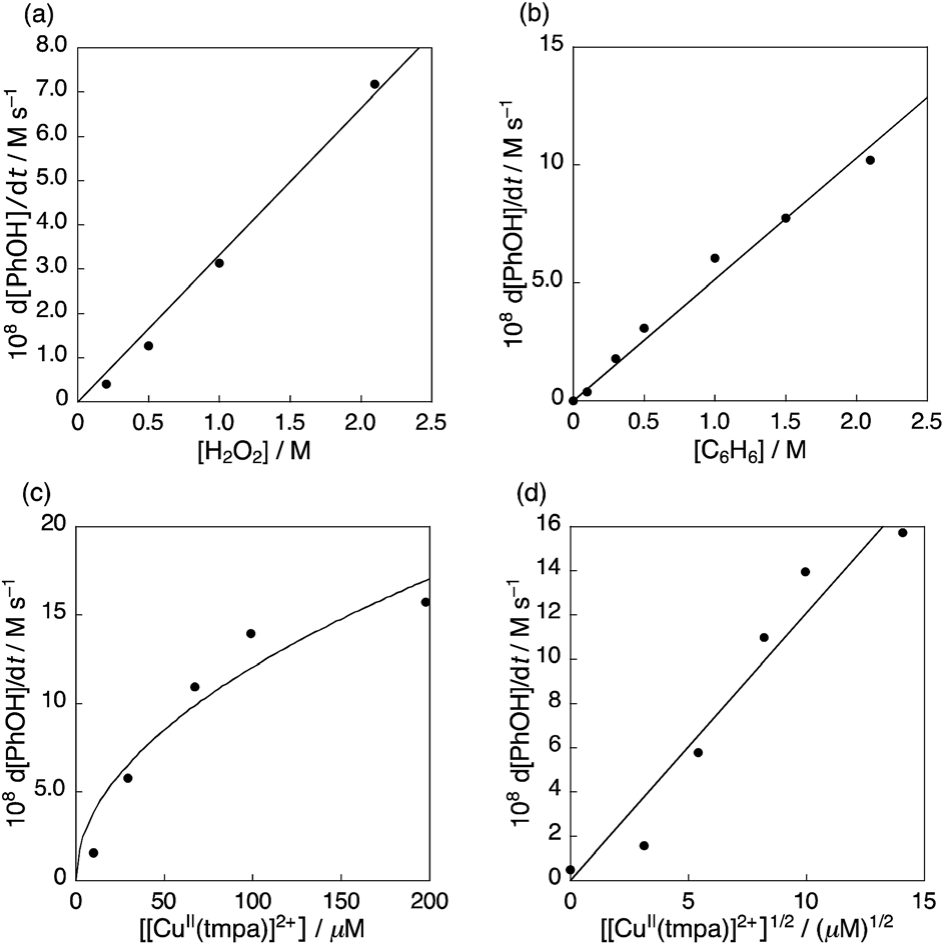
(a) Dependence of the initial rate of formation of phenol (d[PhOH]/d*t*) calculated at 10 h on the concentration of H_2_O_2_ ([H_2_O_2_] = 0–2.1 M, [C_6_H_6_] = 1.0 M). (b) Dependence of d[PhOH]/d*t* calculated at 14 h on the concentration of benzene ([C_6_H_6_] = 0–2.1 M). (c) Dependence of d[PhOH]/d*t* calculated at the time when *ca.* 2 mM of phenol was formed on the concentration of **1** ([[Cu^II^(tmpa)]^2+^] = 0–200 μM). (d) Plot of d[PhOH]/d*t vs.* [[Cu^II^(tmpa)]^2+^]^1/2^. The [PhOH]/d*t* values were determined from the time profiles of formation of phenol in the hydroxylation of benzene with 30 wt% aqueous H_2_O_2_ catalysed by **1** in acetone at 298 K at the same reaction time or when the similar concentration of phenol was produced in the range where the phenol formation rate was constant (Fig. S4†). The concentrations are [C_6_H_6_] = 2.1 M, [H_2_O_2_] = 2.1 M, [H_2_O] = 8.4 M and [[Cu^II^(tmpa)]^2+^] = 67 μM in acetone (4.75 mL) at 298 K unless otherwise noted.

The dependencies of the initial rate of phenol production on the concentrations of **1**, H_2_O_2_ and benzene were examined to determine a kinetic formulation (Fig. 4). The initial rates of phenol production were determined from the time profiles of production of phenol in the catalytic hydroxylation of benzene with H_2_O_2_ (Fig. S4a–c in ESI†). The initial rate of phenol production was proportional to the concentrations of H_2_O_2_ and benzene, as shown in Fig. 4a and b, respectively. The dependence of the initial rate of phenol production on the concentration of **1** was not linear (Fig. 4c), but instead was proportional to the square root of the concentration of **1**. Thus, the kinetic equation for the catalytic hydroxylation of benzene with H_2_O_2_ is given by eqn (2) as follows:

2 d[PhOH]/d*t* = *k*_cat_[**1**]^1/2^[C_6_H_6_][H_2_O_2_]

where *k*_cat_ is the catalytic rate constant. The dependence of the rate on the square root of the concentration of **1** usually suggests that the catalyst may dissociate into two species, which are in equilibrium with **1**, and that the concentration of the two catalytically active species are proportional to the square root of the concentration of **1** when the equilibrium lies to the far left-hand side. However, the Cu(ii) complex (**1**) is a monomer complex, which has no way to dissociate into two species. The unusual kinetic formulation in eqn (2) in terms of the dependence of the rate on **1** provides valuable insight into the catalytic mechanism as discussed later.

### A spin trap in the catalytic hydroxylation of benzene with H_2_O_2_

**Fig. 5:**
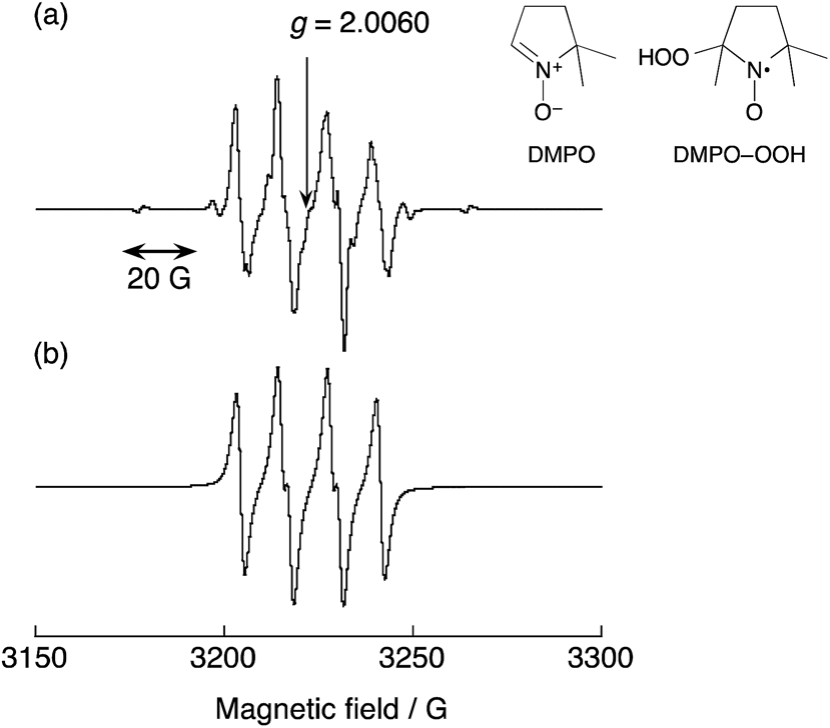
(a) An EPR spectrum of DMPO–OOH produced in an acetone solution of benzene, 30 wt% aqueous H_2_O_2_, **1** and DMPO at 298 K with structures of DMPO and DMPO–OOH. (b) An EPR simulation spectrum of the DMPO–OOH spin adduct. The parameters obtained by the simulation are as follows: *a*_H_ = 10.8 G, *a*_N_ = 13.1 G. The concentrations are [C_6_H_6_] = 2.1 M, [H_2_O_2_] = 0.10 M, [H_2_O] = 0.23 M, [[Cu^II^(tmpa)]^2+^] = 67 μM, [DMPO] = 0.20 M in acetone (2.0 mL).

The trapping of a reactive free radical species with a diamagnetic spin trap to generate a persistent spin adduct, which can be characterised by its EPR spectrum constitutes the well known spin trapping technique.^32^–^46^ 5,5-Dimethyl-1-pyrroline *N*-oxide (DMPO in Fig. 5a) is one of the most popular spin traps, and EPR features of its spin adducts are well established. For example, the EPR spectrum of the HO˙ radical adduct (DMPO–OH) exhibits signals with a relative peak ratio of 1 : 2 : 2 : 1 with the hyperfine coupling constants of *a*_N_ = *a*_H_ = 14.9 G, whereas the EPR spectrum of the HO_2_˙ radical adduct affords more lines with a hyperfine coupling constant of *a*_N_ (13–14 G), which is significantly larger than *a*_H_ (9–11 G).^32^,^37^–^41^,^47^ Carbon-centred radical adducts afford a larger *a*_H_ value than the *a*_N_ value.^32^,^42^ Thus, the type of radical species can be readily distinguished based on the hyperfine splitting pattern of the DMPO spin adduct.

Fig. 5 shows an EPR spectrum observed during the reaction of benzene with H_2_O_2_ in the presence of **1** and DMPO. The hyperfine splitting pattern of the observed radical clearly indicates that HO_2_˙ is produced in the reaction of benzene with H_2_O_2_ and **1**, and is trapped by DMPO to produce DMPO–OOH, which has the *g* value of 2.0060 and *a*_N_ = 13.1 G and *a*_H_ = 10.8 G, as shown in Fig. 5, wherein the observed spectrum (part a) agrees with the computer simulation spectrum (part b). It was confirmed that the addition of DMPO to an acetone solution of benzene and H_2_O_2_ resulted in no observation of an EPR signal. In summary, when **1** was added to the acetone solution of benzene, H_2_O_2_ and DMPO, a strong EPR signal assigned to DMPO–OOH was observed.

**Fig. 6:**
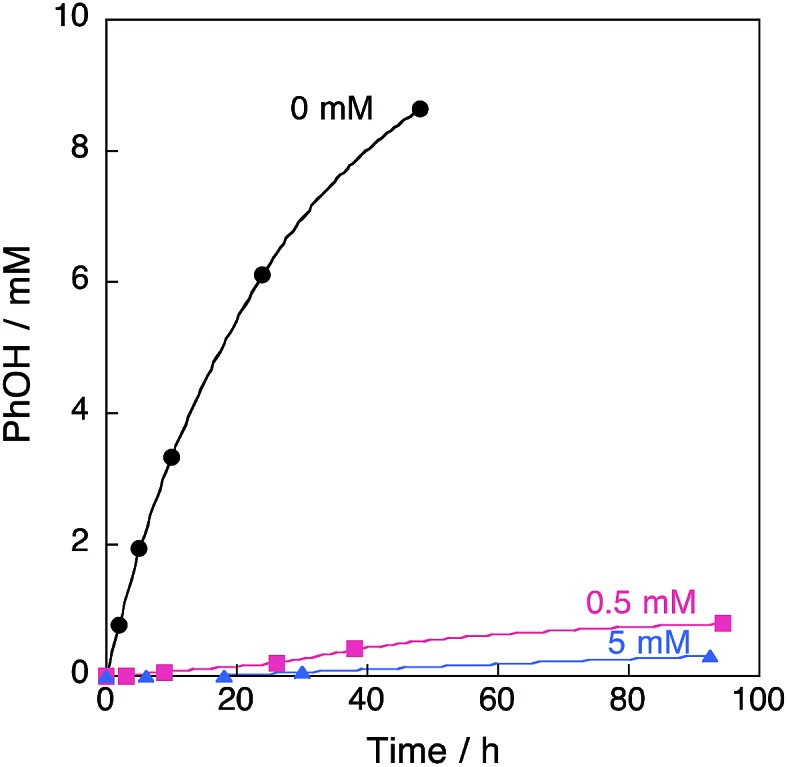
Time profiles of formation of phenol in the reaction of benzene with 30 wt% aqueous H_2_O_2_ in the presence of **1** and DMPO (0, 0.5, 5 mM) in acetone at 298 K. The concentrations are [C_6_H_6_] = 2.1 M, [H_2_O_2_] = 2.1 M, [H_2_O] = 0.4 M and [[Cu^II^(tmpa)]^2+^] = 67 μM (4.75 mL).

**Scheme 1:**
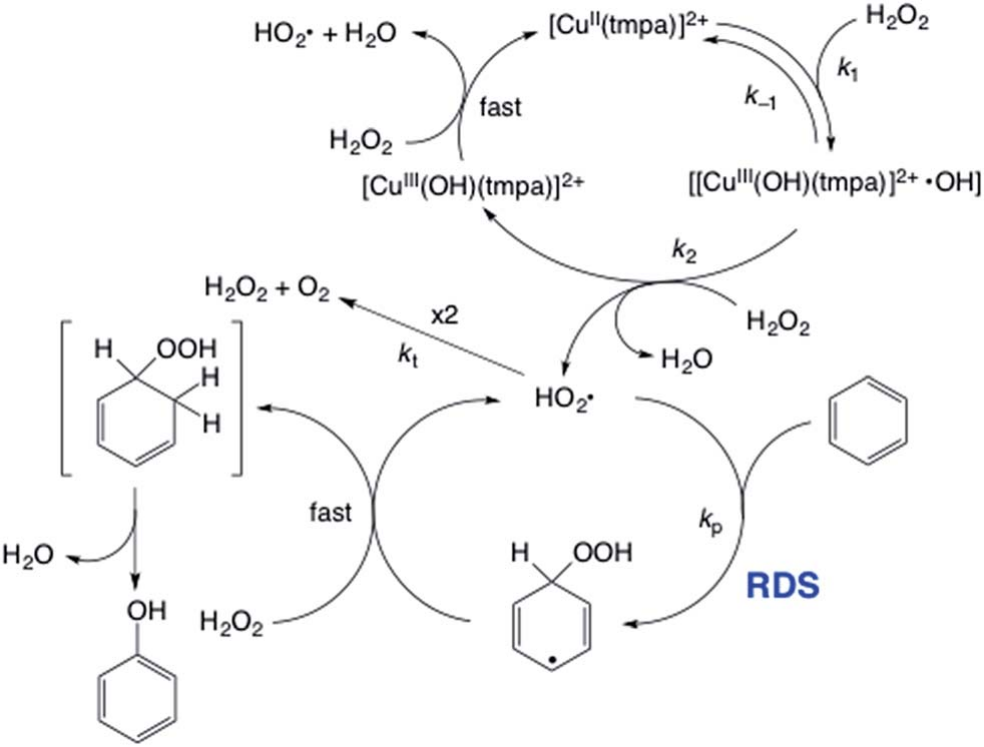
Proposed radical chain mechanism of **1**-catalysed hydroxylation of benzene with H_2_O_2_.

The concentration of DMPO–OOH was determined by comparing the double integration value of the EPR signal of DMPO–OOH with that of a stable reference radical (2,2-diphenyl-1-picrylhydrazyl radical, see Experimental section). The concentration of DMPO–OOH increased with the reaction time, as shown in Fig. S5 (ESI).† More importantly, the addition of a catalytic amount of DMPO to the reaction solution of benzene with H_2_O_2_ and **1** resulted in nearly complete inhibition of the reaction, as shown in Fig. 6. This indicates that HO_2_˙ produced in the reaction of **1** with H_2_O_2_ acts as a chain carrier in the catalytic hydroxylation of benzene, which proceeds *via* radical chain reactions (*vide infra*). The chain length of the propagation step in Scheme 1 is evaluated as the ratio of the rate of formation of phenol (1.08 × 10^–7^ M s^–1^ determined at 5 h) to twice of DMPO–OOH (9.62 × 10^–11^ M s^–1^ determined at 6 h), which is 1120, because two HO_2_˙ radicals are produced in the initiation step (see Scheme 1).^48^

### A radical chain mechanism of the **1**-catalysed hydroxylation of benzene with H_2_O_2_

A proposed radical chain mechanism^48^–^50^ of the **1**-catalysed hydroxylation of benzene with H_2_O_2_, which agrees with the kinetic formulation [eqn (2)], is shown in Scheme 1. The catalytic reaction is started by the Fenton-like reaction,^43^,^44^,^51^ in which H_2_O_2_ is reduced by [Cu^II^(tmpa)]^2+^ to produce HO˙ radical and the hydroxide adduct ([Cu^III^(OH)(tmpa)]^2+^). In competition with the fast back reaction between HO˙ and [Cu^III^(OH)(tmpa)]^2+^, HO˙ reacts with H_2_O_2_ to produce HO_2_˙ and [Cu^III^(OH)(tmpa)]^2+^ also reacts with H_2_O_2_ to produce HO_2_˙, accompanied by regeneration of [Cu^II^(tmpa)]^2+^. HO_2_˙ reacts with benzene to produce the HO_2_˙ adduct of benzene as the rate-determining step (RDS). The subsequent fast hydrogen abstraction from H_2_O_2_ by the HO_2_˙ adduct produces phenol and H_2_O, accompanied by regeneration of HO_2_˙, thus constituting the radical chain reactions. The termination step is the disproportionation reaction of two HO_2_˙ to produce H_2_O_2_ and O_2_. Once HO_2_˙ is trapped by DMPO to produce DMPO–OOH, the radical chain is stopped to inhibit the benzene hydroxylation.

The rate of formation of phenol in Scheme 1 is given by eqn (3) as follows:

3 d[PhOH]/d*t* = *k*_p_[C_6_H_6_][HO_2_˙]

where *k*_p_ is the propagation rate constant of the reaction of HO_2_˙ with benzene. The rate of formation and decay of HO_2_˙ is given by eqn (4) as follows:

4 d[HO_2_˙]/d*t* = 2*k*_2_[([Cu^III^(OH)(tmpa)]^2+^ HO˙)][H_2_O_2_] – 2*k*_t_[HO_2_˙]^2^

where *k*_2_ is the rate constant of the reaction of the caged radical pair ([Cu^III^(OH)(tmpa)]^2+^ HO˙) with H_2_O_2_ to produce HO_2_˙ and *k*_t_ is the rate constant of disproportionation of HO_2_˙. The rate of decay of HO_2_˙ by the reaction with benzene is the same as the rate of formation of HO_2_˙ by the reaction of the HO_2_˙ adduct of benzene with H_2_O_2_. The rate of formation and decay of the caged radical pair ([Cu^III^(OH)(tmpa)]^2+^ HO˙) is given by eqn (5) as follows:

5 d[([Cu^III^(OH)(tmpa)]^2+^ HO˙)]/d*t* = *k*_1_[**1**][H_2_O_2_] – (*k*_–1_ + *k*_2_[H_2_O_2_])[([Cu^III^(OH)(tmpa)]^2+^ HO˙)]

where *k*_1_ is the rate constant of the Fenton-like reduction of H_2_O_2_ by **1** and *k*_–1_ is the rate constant of the back reaction. Under the steady-state conditions (d[HO_2_˙]/d*t* = 0), [HO_2_˙] is given from eqn (4) by eqn (6), whereas [([Cu^III^(OH)(tmpa)]^2+^HO˙)] is given by equation as follows:

6 [HO_2_˙] = (*k*_2_/*k*_t_)^1/2^([([Cu^III^(OH)(tmpa)]^2+^ HO˙)][H_2_O_2_])^1/2^

Under the conditions that *k*_2_[H_2_O_2_] ≪ *k*_–1_, eqn (7) is

7 [([Cu^III^(OH)(tmpa)]^2+^ HO˙)] = *k*_1_[**1**][H_2_O_2_]/(*k*_–1_ + *k*_2_[H_2_O_2_])

reduced to eqn (8). From eqn (3), (6) and (8), the kinetic

8 [([Cu^III^(OH)(tmpa)]^2+^ HO˙)] = *k*_1_[**1**][H_2_O_2_]/*k*_–1_

formulation is derived as given by eqn (9), which agrees with

9 d[PhOH]/d*t* = *k*_p_(*k*_1_/*k*_–1_)^1/2^(*k*_2_/*k*_t_)^1/2^[**1**]^1/2^[C_6_H_6_][H_2_O_2_]

the experimental observations (eqn (2)). Such agreement, including the unusual dependence of the rate on [**1**] strongly supports the validity of Scheme 1. The feasibility of the chain reactions in Scheme 1 is also supported by DFT calculations (see Fig. S6, Tables S1 and S2 in ESI†).^52^ The rate-determining propagation step of the reaction of HO_2_˙ with benzene to produce the HO_2_˙ adduct of benzene is calculated to be uphill by 11.1 kcal mol^–1^, whereas the reaction of the HO_2_˙ adduct of benzene with HO_2_˙ to produce phenol and H_2_O is downhill by 72.3 kcal mol^–1^. The formation of the intermediate prior to phenol and H_2_O is uphill by 5.6 kcal mol^–1^. Thus, the intermediate is given in parenthesis in Scheme 1.

### Incorporation of Cu(ii) complex catalyst **1** in a mesoporous silica–alumina

Mesoporous silica and silica–alumina have often been used to improve the catalytic performance of metal complexes in a role as catalyst supports whose pores provide broad reaction fields with efficient incorporation.^15^,^16^,^21^,^26^,^53^–^57^ To improve the reaction selectivity of the benzene hydroxylation by H_2_O_2_, the catalyst **1** was incorporated into a mesoporous silica–alumina Al-MCM-41 (ref. 53*a*, 54–56) (the Brunauer–Emmett–Teller (BET) surface area: 1200 m^2^ g^–1^, the external surface area: 32 m^2^ g^–1^, the pore diameter: 3.5 nm) to prepare [Cu^II^(tmpa)]^2+^@Al-MCM-41 (see Experimental section).^26^,53a,^58^ [Cu^II^(tmpa)]^2+^@Al-MCM-41 was characterised by a UV-Vis diffuse reflectance spectrum (Fig. S7 in ESI†) and the amount of incorporated **1** was determined to be 2.50 × 10^–5^ mol g^–1^ by the absorption spectral change of the mother liquid according to the literature (Fig. S8 in ESI†).53*a* The much larger BET surface area compared to the external surface suggests that most of the incorporated [Cu(tmpa)]^2+^ is placed inside the mesopore, which is large enough (diameter 3.5 nm) for incorporation of [Cu(tmpa)]^2+^ species smaller than 1.2 nm.

**Fig. 7:**
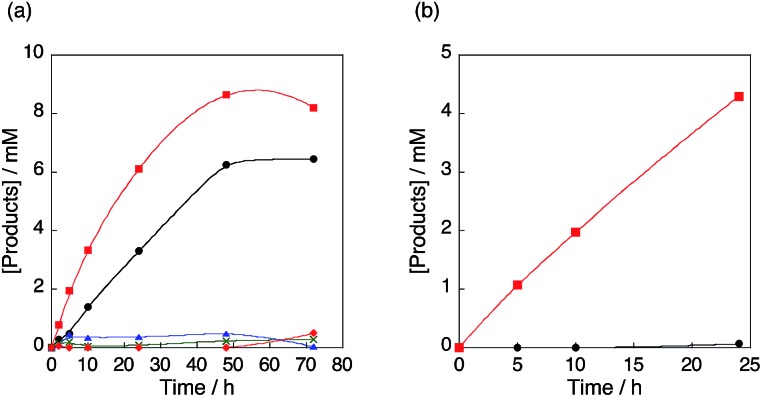
(a) Time profiles of formation of phenol in the hydroxylation of benzene with 30 wt% aqueous H_2_O_2_ and a catalytic amount of **1** (red square), [Cu^II^(tmpa)]^2+^@Al-MCM-41 (12.5 mg, black circle), Al-MCM-41 (12.5 mg, blue triangle) or no Cu(ii) species nor Al-MCM-41 (green cross), and formation of *p*-benzoquinone in the hydroxylation of benzene with **1** (red diamond) in acetone at 298 K. (b) Time profiles of formation of *p*-benzoquinone in hydroxylation of phenol with 30 wt% aqueous H_2_O_2_ and a catalytic amount of [Cu^II^(tmpa)]^2+^@Al-MCM-41 (12.5 mg, black circle) or [Cu^II^(tmpa)]^2+^ (red square) in acetone at 298 K. The concentrations are [C_6_H_6_] or [PhOH] = 2.1 M, [H_2_O_2_] = 2.1 M and [[Cu^II^(tmpa)]^2+^] = 67 μM in acetone (4.75 mL).

When [Cu^II^(tmpa)]^2+^@Al-MCM-41 was used as a catalyst for the hydroxylation of benzene with H_2_O_2_, benzene was oxidised to phenol as in the case of the homogeneous system (Fig. 7a).^59^ A slower phenol formation with [Cu^II^(tmpa)]^2+^@Al-MCM-41 compared to that with [Cu^II^(tmpa)]^2+^ can be explained by the smaller diffusion rate in the pore. The unchanged blue colour of [Cu^II^(tmpa)]^2+^@Al-MCM-41 after the reaction suggests that [Cu^II^(tmpa)]^2+^ cations are encapsulated in Al-MCM-41 without major leaching. The selectivity to produce phenol was significantly improved as *p*-benzoquinone was hardly produced from phenol, as shown in Fig. 7b. It was confirmed that no phenol was produced using Al-MCM-41 without **1**, indicating that the reaction was catalysed by [Cu^II^(tmpa)]^2+^ incorporated into Al-MCM-41 (Fig. 7a). It was found that benzene does adsorb to Al-MCM-41, whereas phenol was hardly adsorbed (Fig. S9 and S10 in the ESI†). The difference in the adsorption behaviours can be explained by the solvophobic surface present in Al-MCM-41 compared to the environment in solution.^60^ This selective adsorption of benzene and lack of adsorption of phenol results in the selective hydroxylation of benzene with H_2_O_2_ catalysed by the [Cu^II^(tmpa)]^2+^ sites in Al-MCM-41 to produce only phenol, which is desorbed from Al-MCM-41. The solution was not coloured after the reaction, suggesting that leaching of the Cu^2+^ species is not the major reason for the saturation of phenol formation. This result shows the potential for recyclability of [Cu^II^(tmpa)]^2+^@Al-MCM-41 after recovery and washing with acetonitrile.

**Fig. 8:**
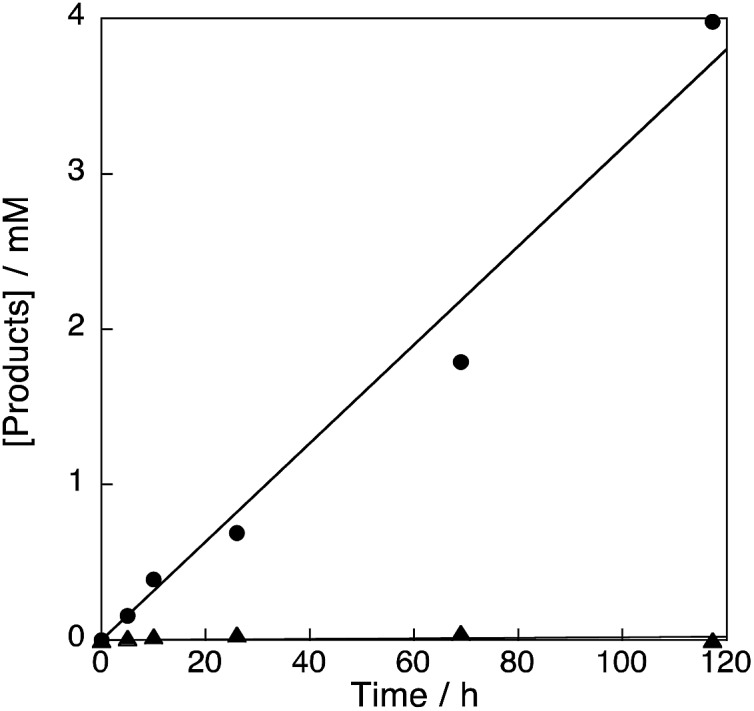
Time profiles of formation of phenol (circle) and *p*-benzoquinone (triangle) in the hydroxylation of benzene (2.1 M) with 30 wt% aqueous H_2_O_2_ (2.1 M) catalysed by [Cu^II^(tmpa)]^2+^@Al-MCM-41 (1.0 μM) in acetone (4.75 mL) at 298 K.

The reaction of 1 mM benzene with 2.1 M H_2_O_2_ and 200 μM of [Cu^II^(tmpa)]^2+^@Al-MCM-41 resulted in formation of phenol in 17% yield with 100% selectivity after 118 h (Fig. S11 in ESI†). The durability of the catalyst [Cu^II^(tmpa)]^2+^@Al-MCM-41 was also examined by the reaction of 2.1 M benzene with 2.1 M H_2_O_2_ and 1 μM of the catalyst. The turnover number was determined to be 4320 after 118 h, demonstrating a high durability for this catalyst (Fig. 8).

## Conclusions

In conclusion, one-step hydroxylation of benzene with H_2_O_2_ to produce phenol with a high turnover number (TON = 4320) and high selectivity (>99%) has been achieved using a copper(ii) complex, ([Cu(tmpa)]^2+^), incorporated into mesoporous silica–alumina (Al-MCM-41) in acetone at 298 K. The catalytic hydroxylation of benzene with H_2_O_2_ proceeds *via* a radical chain mechanism in which HO_2_˙ produced *via* the Fenton-like reduction of H_2_O_2_ with [Cu(tmpa)]^2+^ acts as the chain carrier radical. Spin trapping of the chain carrier radical resulted in the complete inhibition of the catalytic benzene hydroxylation. The present study on the selective catalytic hydroxylation of benzene to phenol using metal complex-incorporated mesoporous silica–alumina as a catalyst provides a general strategy that may be applied to other selective catalytic reactions.

## Experimental section

### Materials

Chemicals were purchased from commercial sources and used without further purification, unless otherwise noted. Tetraethyl orthosilicate, cetyltrimethylammonium bromide, sodium aluminate, sodium hydroxide, aqueous solutions of hydrogen peroxide (30 wt%) and perchloric acid (60%) were purchased from Wako Pure Chemical Industries. Tris(2-pyridylmethyl)amine and oxo[5,10,15,20-tetra(4-pyridyl)porphyrinato]titanium(iv) (Ti–TPyP) were supplied from Tokyo Chemical Industry. Copper(ii) perchlorate hexahydrate was supplied by Sigma-Aldrich. Acetone and acetonitrile were purchased from Nacalai tesque as a spectral grade and used as received. Ultra-pure water was provided by a water purification system, Millipore Direct-Q3 UV, wherein the electronic conductance was 18.2 MΩ cm. Benzene was purchased from Wako Pure Chemical Industries and purified by washing with sulphuric acid and water following distillation. [Cu(tmpa)(CH_3_CN)](ClO_4_)_2_, [Cu(tepa)(ClO_4_)]ClO_4_, [Cu^II^_2_(N5)(H_2_O)_2_](NO_3_)_4_ and Al-MCM-41 were synthesised by literature methods.53a,^61^–^64^

### Synthesis of a mesoporous silica–alumina Al-MCM-41

Al-MCM-41 was synthesised by a reported method.^64^ Cetyltrimethylammonium bromide (18.3 g, 50.4 mmol) was dissolved in an aqueous solution (1.0 L) of NaOH (8.39 g, 210 mmol). Tetraethylorthosilicate (87.5 g, 94.0 mL, 420 mmol) was added dropwise to the aqueous solution during 3 h at 35–40 °C with stirring in water bath and stirred for a further 30 min. Then, sodium aluminate (0.484 g, 5.90 mmol) was added to the reaction vessel and stirred for 4 h at 298 K. The white precipitate obtained was filtered under reduced pressure and washed with distilled water. After drying at 333 K in an oven for 10 h, the solid obtained was calcined at 873 K for 6 h at a rate of 1 K min^–1^ of temperature increase. The Si/Al ratio of this solid is 60,^65^ which was calculated from the amount of the precursor materials. The Brunauer–Emmett–Teller (BET) surface area and the external surface area of Al-MCM-41 were determined to be 1200 m^2^ g^–1^ and 32 m^2^ g^–1^, respectively, by the N_2_ isotherm and the *t*-plot. The pore diameter was also determined to be 3.5 nm from the powder XRD pattern. Al-MCM-41 with the ratio of Si/Al = 20 was prepared by the same method with the appropriate amount of sodium aluminate.

### Preparation of [Cu^II^(tmpa)]^2+^@Al-MCM-41

[Cu^II^(tmpa)]^2+^@Al-MCM-41 was prepared by a cation exchange method in an acetonitrile solution according to the literature method.^26^ Al-MCM-41 (300 mg) was suspended in an acetonitrile solution (15 mL) of [Cu^II^(tmpa)(CH_3_CN)](ClO_4_)_2_ (16.5 mg, 27.8 μmol, [[Cu^II^(tmpa)]^2+^]: 1.85 mM) and stirred for 2 h at 298 K. The suspension was centrifuged to collect solid, which was washed with acetonitrile (5.0 mL × 2) and dried *in vacuo*. Incorporation of [Cu^II^(tmpa)]^2+^ was confirmed by the diffuse reflectance spectrum of the solid obtained resembling the absorption spectrum of [Cu^II^(tmpa)]^2+^ in acetonitrile (Fig. S7†),^26^,53a which could also be observed by clear colour change of the solid from white to blue. The amount of incorporated [Cu^II^(tmpa)]^2+^ was determined to be 2.50 × 10^–5^ mol g^–1^ by the decrease in the UV-Vis absorption band around 860 nm due to [Cu^II^(tmpa)]^2+^ in the mother liquid according to the literature (Fig. S8†).53*a*

### The catalytic hydroxylation reaction

A typical procedure for catalytic hydroxylation is as follows: 4.75 mL of an acetone solution containing benzene (2.1 M) and 30 wt% aqueous H_2_O_2_ (2.1 M) was added to [Cu^II^(tmpa)(CH_3_CN)](ClO_4_)_2_ (0.31 μmol) and vigorously stirred at 298 K. The sample solutions for GC-MS measurements were prepared from the reaction solution by dilution with acetonitrile as needed to analyse products. The Al-MCM-41 and [Cu^II^(tmpa)]^2+^@Al-MCM-41 were removed by filtration before the measurement. GC-MS measurements were performed with a Shimadzu QP-2010 Ultra instrument.

### Electrochemical measurements

Cyclic voltammetry (CV) measurements were performed with an ALS630B electrochemical analyser in deuterated acetonitrile containing 0.1 M Bu_4_NPF_6_ (TBAPF_6_) as a supporting electrolyte at 298 K. The platinum working electrode (BAS, surface i.d. 1.6 mm) was polished with BAS polishing alumina suspension and rinsed with Milli-Q ultra-pure water, acetone and acetonitrile before use. The counter electrode was a platinum wire (0.5 mm dia.). The measured potentials were recorded with respect to an Ag/AgNO_3_ (0.01 M) reference electrode. The values of redox potentials (*vs.* Ag/AgNO_3_) are converted into those *vs.* SCE by addition of 0.29 V.^66^

### Titanium(iv)–porphyrin method

The concentration of hydrogen peroxide was determined using the Ti–TPyP reagent according to the literature as follows:^31^ aqueous solutions of 50 μM Ti–TPyP with 50 mM hydrochloric acid and 5 M perchloric acid were prepared. Sample solutions were prepared by 5000 times dilution of 50 μL of the reaction mixture (the concentrations are [C_6_H_6_] = 2.1 M, [H_2_O_2_] = 0.6 M and [[Cu^II^(tmpa)]^2+^] = 200 μM in acetone (4.75 mL)) with acetonitrile. The sample solution (50 μL) was added to the mixed solution of Ti–TPyP (250 μL) and perchloric acid (250 μL), shaken and diluted with H_2_O (1950 μL). The concentration of hydrogen peroxide was calculated from the absorbance of the resulting peroxo complex at 435 nm observed by UV-Vis measurements (Hewlett-Packard 8453 diode array spectrophotometer).

### Spin trap by DMPO and EPR measurements

A typical procedure for a spin trap experiment is as follows: 4.75 mL of an acetone solution containing benzene (2.1 M), 30 wt% aqueous H_2_O_2_ (0.1 M), **1** (0.31 μmol, 67 μM), and DMPO (0.2 M) was vigorously stirred at 298 K. The sample for EPR was prepared by Ar bubbling (15 min) with a Teflon tube of 200 μL of the reaction solution in a quartz EPR tube (2.0 mm i.d.). EPR spectra of solutions were obtained on a JEOL X-band spectrometer (JES-RE1XE) at 298 K. The *g* value was calibrated using a Mn^2+^ marker. The EPR spectra were obtained under non-saturating microwave power conditions. The magnitude of modulation was chosen to optimise the resolution and the signal-to-noise (S/N) ratio of the observed spectra. The double integrated first derivative of a stable radical, 2,2-diphenyl-1-picrylhydrazyl (DPPH) radical (10 μM), was obtained from an EPR spectrum in a mixed solvent benzene/acetone/H_2_O (1 : 1 : 5, v/v/v) similar to the reaction conditions and used as a reference. The spin concentrations of DMPO–OOH signals in the reaction solutions observed by EPR were calculated by comparing with the value obtained from DPPH. The GC-MS (Shimadzu QP-2010 Ultra) measurements were performed as needed to examine the relationship between the spin trap and the hydroxylation.

### UV-Vis absorption spectra measurements

UV-Vis absorption spectra were obtained on a Hewlett-Packard 8453 diode array spectrophotometer for UV-Vis at 298 K. UV-Vis diffuse reflectance spectra were obtained on a JASCO V-670 spectrophotometer equipped with an SIN-768 attachment.

### Theoretical calculations

Density functional theory (DFT) calculations were performed on a 32CPU workstation (PQS, Quantum Cube QS8-2400C-064). Geometry optimisations were carried out using the B3LYP/6-31++G(d) level of theory for compounds and intermediates relating to the reaction as implemented in the Gaussian 09 program, Revision A.02.^52^

## Supplementary Material

Supplementary information
