# Supplementary material for: One-step selective hydroxylation of benzene to phenol with hydrogen peroxide catalysed by copper complexes incorporated into mesoporous silica–alumina
Source: Chem Sci. 2016 Jan 5;7(4):2856–63. doi: 10.1039/c5sc04312c (PMC4951108; doi:10.1039/c5sc04312c)
Supplement: Supplementary file 1 [file SC-007-C5SC04312C-s001.pdf]

Electronic Supplementary Material (ESI) for Chemical Science.
This journal is © The Royal Society of Chemistry 2016

Electronic Supplementary Information

One-Step Selective Hydroxylation of Benzene to Phenol with Hydrogen Peroxide Catalysed by Copper Complexes Incorporated into Mesoporous Silica- Alumina

Mihoko Yamada,[a] Kenneth D. Karlin*,[b] and Shunichi Fukuzumi*,[acd]

[a] Dr. M. Yamada, Prof. Dr. S. Fukuzumi

Department of Material and Life Science, Graduate School of Engineering, Osaka University, ALCA and SENTAN, Japan Science and Technology (JST), Suita, Osaka 565-0871 (Japan)

Fax: (+81) 6-6879-7370

E-mail: fukuzumi@chem.eng.osaka-u.ac.jp

[b] Prof. Dr. K. D. Karlin

Department of Chemistry, The Johns Hopkins University, Baltimore, Maryland, 21218, (United States)

[c,d] Prof. Dr. S. Fukuzumi

Department of Chemistry and Nano Science, Ewha Womans University, Seoul 120-750 (Korea)

Faculty of Science and Engineering, Meijo University, ALCA and SENTAN, Japan Science and Technology Agency (JST), Nagoya, Aichi 468-0073 (Japan)

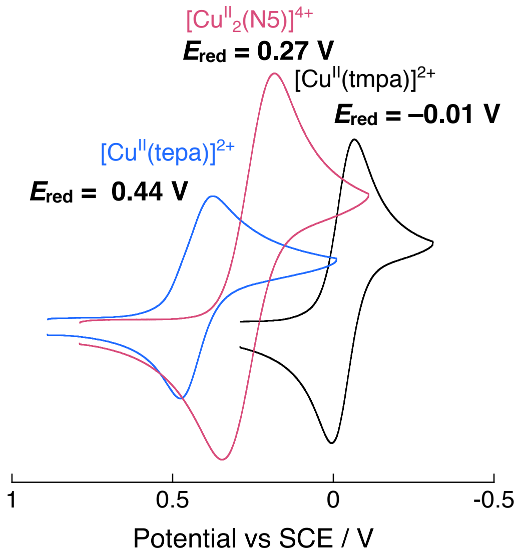

Figure S1. Cyclic voltammogram of [Cu^{II}(tmpa)(CH_3CN)](ClO_4)$_2$ (1.0 mM), [Cu$^{II}_2$(N5)(H_2O)$_2$](NO_3)$_4$ (1.0 mM), and [Cu^{II}(tepa)(ClO_4)](ClO_4) (1.0 mM) in deaerated CH_3CN containing *n*-Bu_4NPF_6 (0.10 M) with a platinum disk electrode (i.d. 1.6 mm) at 298 K. Scan rate: 100 mV s^{-1}.

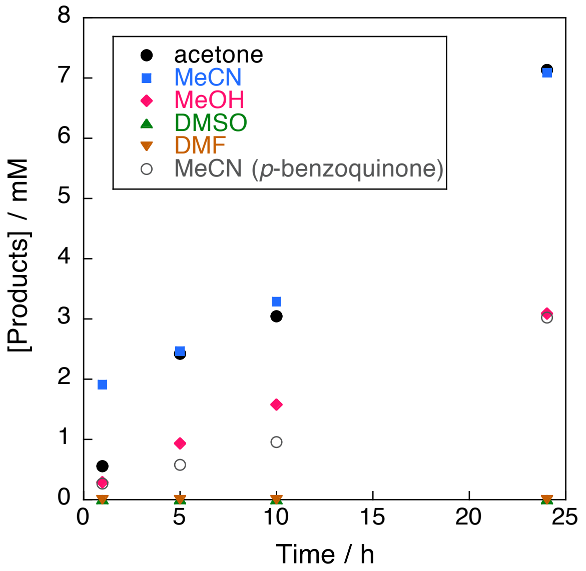

Figure S2. Time profiles of formation of phenol or *p*-benzoquinone in the hydroxylation of benzene with 30 wt% aqueous H_2O_2 and a catalytic amount of **1** at 298 K in various solvents (4.75 ml). Symbols correspond as follows: Solid circle, acetone; solid square, acetonitrile; pink diamond, MeOH; green triangle, DMSO; orange inverted triangle, DMF; grey open circle, acetonitrile (*p*-benzoquinone). The concentrations are [C_6H_6] = 2.1 M, [H_2O_2] = 2.1 M and [[Cu^{II}(tmpa)]$^{2+}$] = 67 μM in 4.75 ml of the starting solutions.

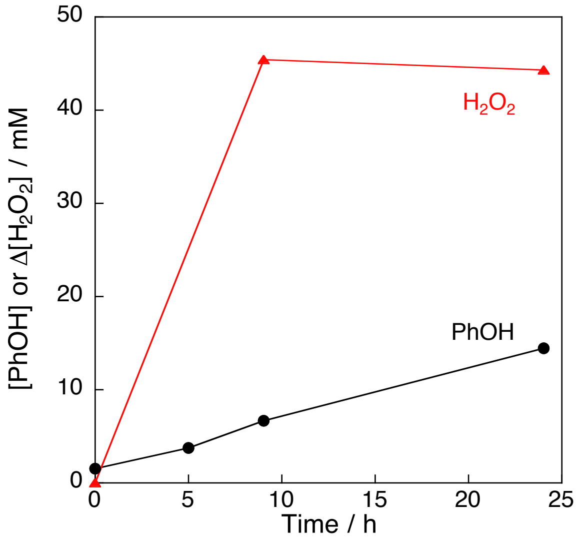

Figure S3. Time profiles of formation of phenol (black circle) and consumption of H_2O_2 (red triangle) in the hydroxylation of benzene with 30 wt% aqueous H_2O_2 with a Cu(II) complex catalyst **1** in acetone at 298 K. The concentrations are $[C_6H_6]$ = 2.1 M, $[H_2O_2]$ = 0.6 M and $[[Cu^{II}(tmpa)]^{2+}]$ = 200 μM in 4.75 ml of the starting solution.

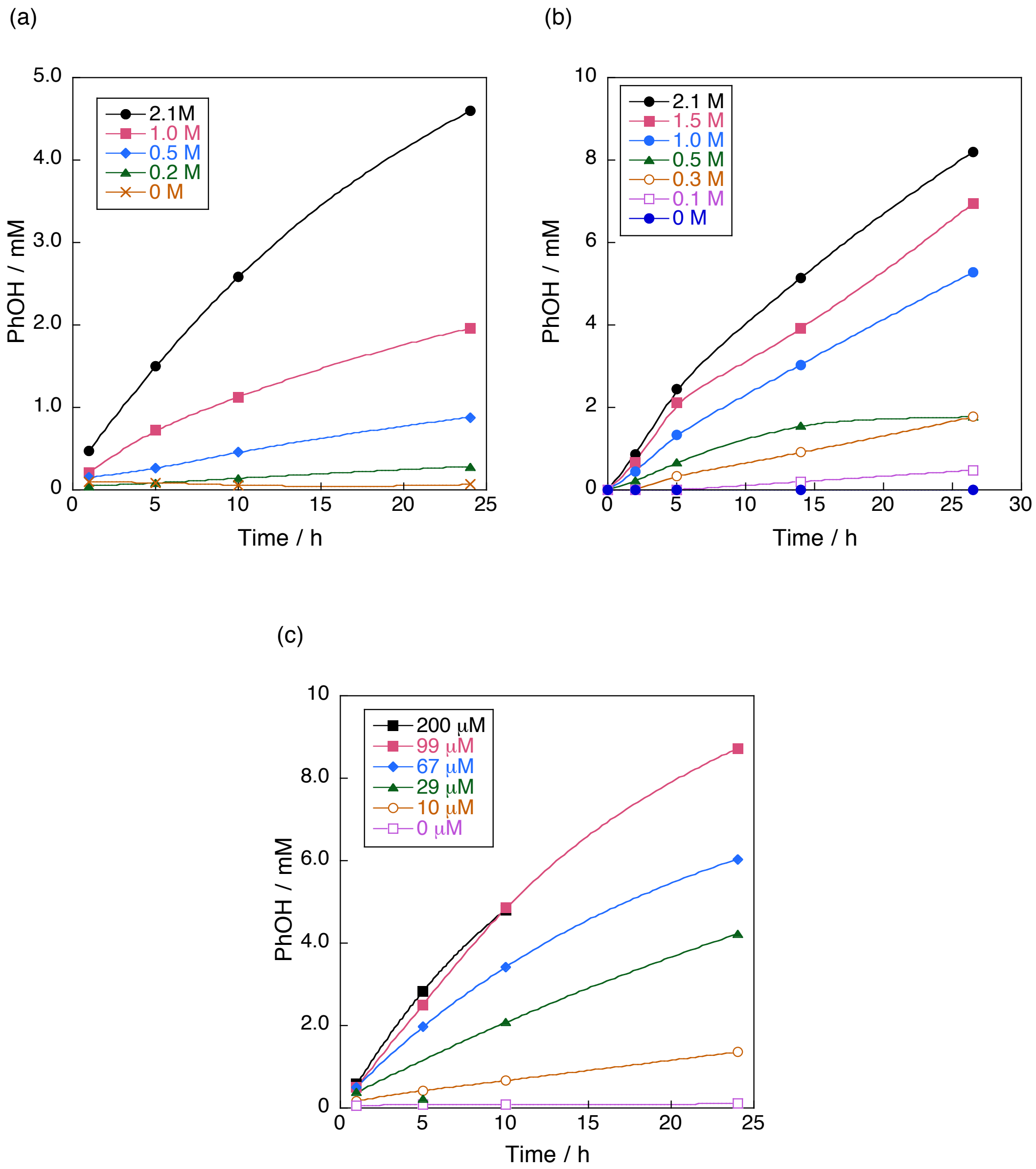

Figure S4. Time profiles of formation of phenol in the hydroxylation of benzene (a) with various amounts of H_2O_2 (0, 0.2, 0.5, 1.0, 2.1 M) with **1** and 1.0 M C_6H_6, (b) from various amounts of benzene (0, 0.1, 0.3, 0.5, 1.0, 1.5, 2.1 M) with H_2O_2 and with **1**, and (c) with various amounts of **1** (0, 10, 29, 67, 99, 200 μM) The concentrations are $[C_6H_6]$ = 2.1 M, $[H_2O_2]$ = 2.1 M, $[H_2O]$ = 8.4 M and $[[Cu^{II}(tmpa)]^{2+}]$ = 67 μM in acetone (4.75 ml) at 298 K unless otherwise noted.

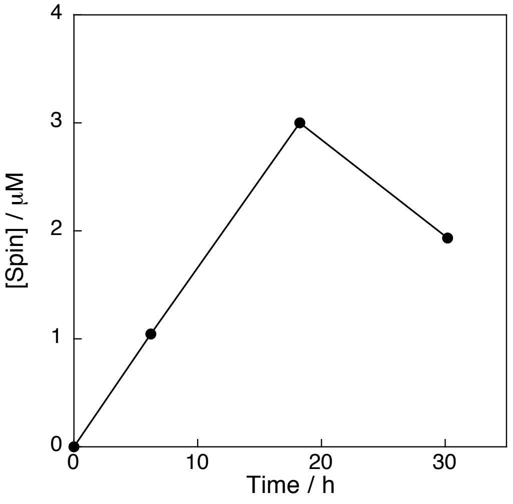

Figure S5. Time profiles of the spin concentration determined by comparing with a reference 2,2-diphenyl-1-picrylhydrazyl radical produced in an acetone solution of benzene, 30 wt% aqueous H_2O_2 and **1** with DMPO at 298 K. The concentrations are $[C_6H_6]$ = 2.1 M, $[H_2O_2]$ = 2.1 M, $[H_2O]$ = 0.4 M, $[[Cu^{II}(tmpa)]^{2+}]$ = 67 μM and [DMPO] = 5.4 mM in acetone (4.75 ml).

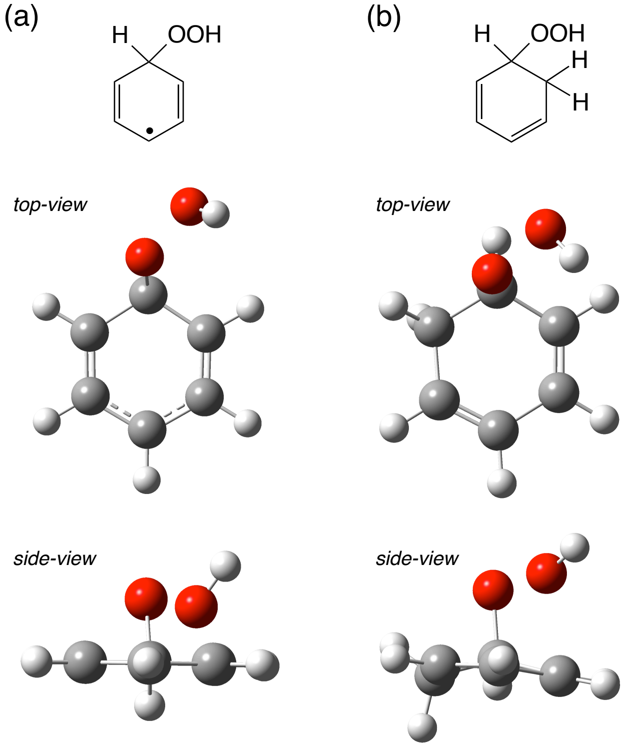

Figure S6. Structural formulas and optimised structures of (a) an OOH-adduct radical and (b) hydrogen-transferred OOH-adduct transition state calculated by a DFT method at the B3LYP/6-31++G(d) level.

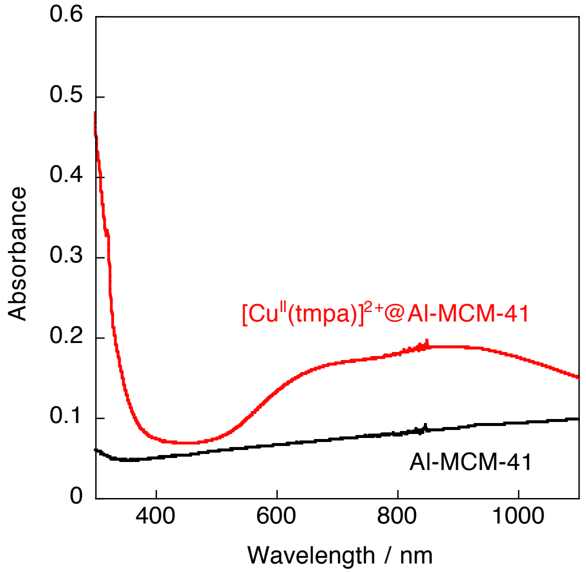

Figure S7. UV-vis diffuse reflectance spectra of [Cu(tmpa)]$^{2+}$@Al-MCM-41 (red line) and Al-MCM-41 (black line)

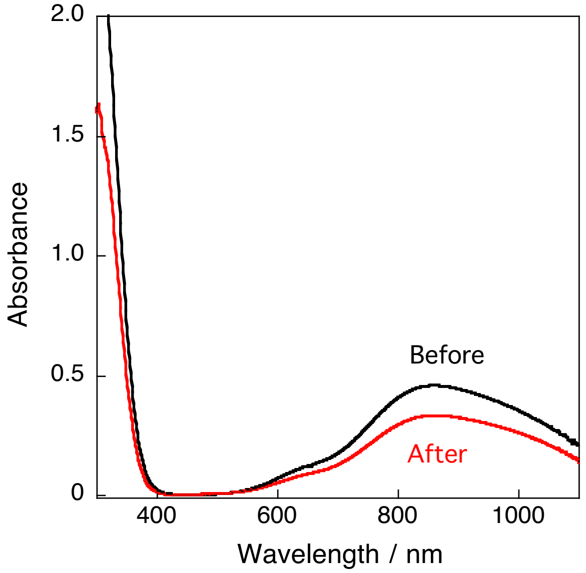

Figure S8. UV-vis absorption spectra of the mother liquid containing [Cu(tmpa)]$^{2+}$ before and after stirring with Al-MCM-41 (300 mg) in acetonitrile at 298 K. The initial conditions are [[Cu(tmpa)]$^{2+}$] = 1.85 mM in acetonitrile (15 ml) at 298 K.

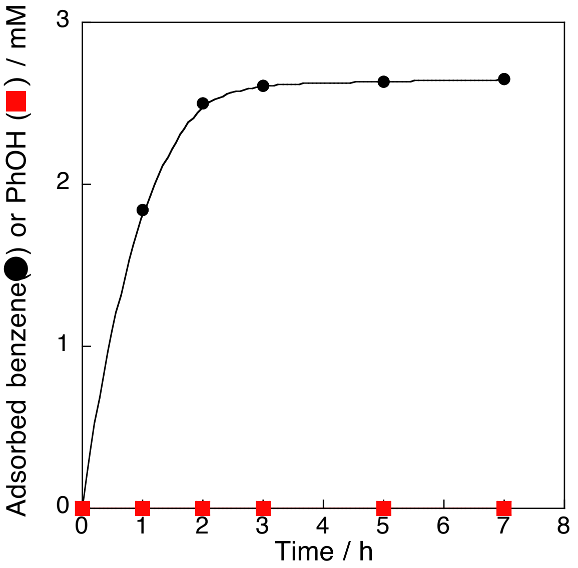

Figure S9. Adsorbed benzene and PhOH by Al-MCM-41 (100 mg) in 4.75 ml of an aqueous solution of benzene (5.1 mM) and PhOH (5.0 mM).

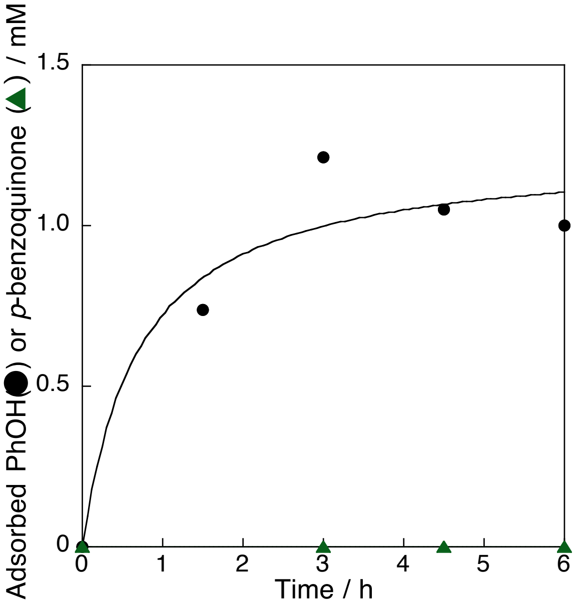

Figure S10. Adsorbed benzene by Al-MCM-41 (50 mg) in 5.0 ml of an acetone/H_2O 4:1 mixed solution of benzene (5.0 mM), and adsorbed PhOH by Al-MCM-41 (50 mg) in 5.0 ml of an acetone/H_2O 4:1 mixed solution of PhOH (5.0 mM). The concentration is [H_2O] = 8.4 M is the same as found in the reaction conditions.

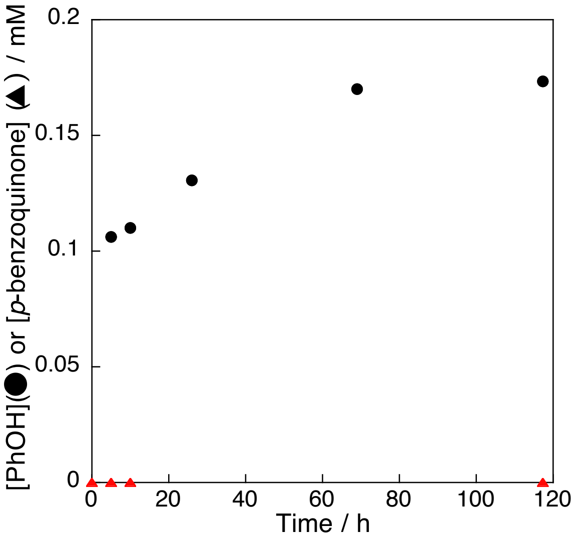

Figure S11. Time profiles of formation of phenol (black solid circle) and *p*-benzoquinone (red solid triangle) in the hydroxylation of benzene with 30 wt% aqueous H_2O_2 and $[Cu^{II}(tmpa)]^{2+}$@Al-MCM-41 at 298 K in acetone (4.75 ml). The concentrations are $[C_6H_6]$ = 1.0 mM, $[H_2O_2]$ = 2.1 M and $[[Cu^{II}(tmpa)]^{2+}]$ = 200 μM.

Table S1. Energy of compounds calculated by DFT method at the B3LYP/6-31++G(d) level.

Compounds	Energy (au)	Compounds	Energy (au)
1	−150.9105	5	−383.7789
2	−232.2590	6	−76.4227
3	−383.1519	7	−307.4805
4	−151.5465		

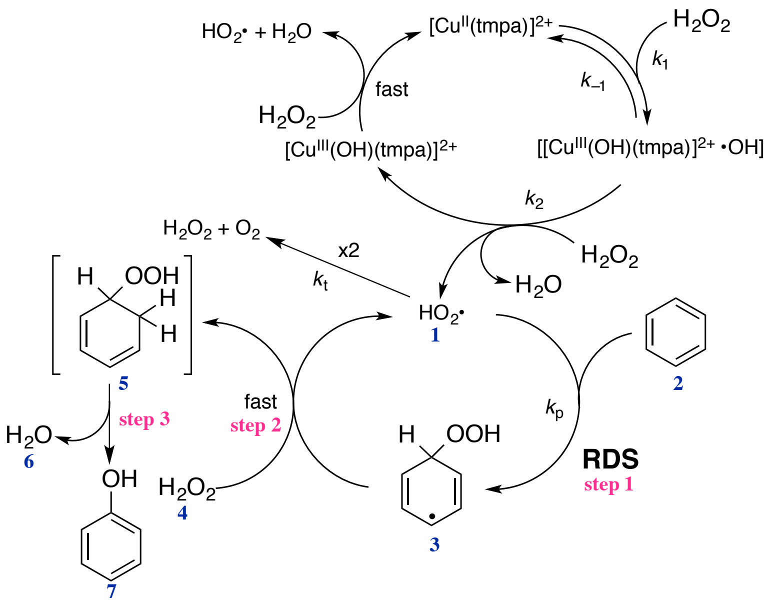

Table S2. Stabilisation energy of each step calculated from results of DFT calculations (1 au = 627.509 kcal mol^{-1}).

Step	Initial state (kcal mol^{-1})	Final state (kcal mol^{-1})	Stabilisation energy (kcal mol^{-1})
1	−240442.5	−240431.4	−11.1
2	−335528.3	−335522.7	−5.6
3	−240824.92	−240902.9	77.9
